# Ultrasound Classification of Thyroid Nodules: A Systematic Review

**DOI:** 10.7759/cureus.7239

**Published:** 2020-03-11

**Authors:** Rakesh Mistry, Christopher Hillyar, Anjan Nibber, Thushanth Sooriyamoorthy, Nirmal Kumar

**Affiliations:** 1 Otolaryngology, Imperial College Healthcare NHS Trust, London, GBR; 2 Surgery, Barts and the London School of Medicine, Barts Health NHS Trust, London, GBR; 3 Neurology, Oxford University Medical School, Oxford University Hospitals NHS Foundation Trust, Oxford, GBR; 4 Surgery, Walsall Healthcare NHS Trust, Birmingham, GBR; 5 Otolaryngology, Wrightington, Wigan and Leigh NHS Foundation Trust, Wigan, GBR

**Keywords:** thyroid, nodules, ultrasound, fine needle aspiration biopsy, tirads, ata, bethesda, thyroid cancer

## Abstract

Ultrasound (US) based classification systems exist for the stratification of thyroid nodules based on the risk for malignancy. This systematic review aimed to assess the evidence for the performance of US-based thyroid nodule classification systems through correlation with fine needle aspiration biopsy (FNAB). PubMed and Scopus were searched using keywords that included ‘ultrasound classification’, ‘thyroid nodules’, ‘fine needle aspiration’, and ‘malignancy’. Inclusion criteria were as follows: studies/reviews reporting on US imaging for the classification of thyroid nodules. Exclusion criteria were as follows: no comparison between US imaging findings and histology reports based on FNAB, no full English text available/accessible. The database searches identified 66 publications. After evaluation, 12 studies met the inclusion criteria. Two US-based classification systems for thyroid nodules were assessed: the Thyroid Imaging Reporting and Data System (TIRADS) and the American Thyroid Association (ATA) guidelines. For TIRADS, the sensitivity, specificity, positive predictive value (PPV), and negative predictive value (NPV) ranged from 70.6% to 97.4%, 29.3% to 90.4%, 23.3% to 64.3%, and 87.1% to 99.0%, respectively. The median sensitivity, specificity, PPV, and NPV for TIRADS was 90.0%, 57.4%, 49.0%, and 91.0%, respectively. One study comparing TIRADS with the ATA guidelines demonstrated that TIRADS was superior in terms of sensitivity, whereas the ATA guidelines were superior in terms of specificity and PPV. The high sensitivity and NPV of the US-based TIRADS classification system have excellent utility for correctly classifying nodules as positive for malignant disease and for predicting the absence of malignant disease. The paucity of studies assessing the ATA guidelines highlights avenues for further research comparing TIRADS with other systems of thyroid nodule classification.

## Introduction and background

High-resolution ultrasound (US) is the gold standard test for the identification of thyroid nodules. Despite the high prevalence of thyroid nodules (up to 12% of adults), the incidence of thyroid cancer is relatively low (3.2 per 100,000) [1]. Although the majority of thyroid nodules are asymptomatic, it is recommended that all patients with palpable nodules undergo US imaging to determine whether the nodule requires a fine needle aspiration biopsy (FNAB), US follow-up, or no further evaluation. However, because of a lack of correlation between clinical symptoms and malignancy, the American Association of Clinical Endocrinologists recommends that all nodules smaller than 10 mm or any suspicious nodules on US imaging should be investigated further using FNAB. This recommendation is based on studies that established that prognosis is inversely related to nodule size [2,3].

Recently, guidelines have been developed in order to permit US imaging to be used for the identification and stratification of nodules based on the risk of malignancy [4-10]. These guidelines include the Thyroid Imaging Reporting and Data System (TIRADS), which was developed and validated based on existing multi-institutional data and expert opinion [11]. The risk stratification of thyroid nodules not only serves to identify patients that require FNAB but also reduces the unnecessary risk and cost associated with performing invasive procedures, such as FNAB, in patients with low-risk nodules that require either US follow-up or no further investigation. Therefore, the decision to perform FNAB should be based on the risk of malignancy rather than the size of the nodule per se.

Currently, multiple systems are used worldwide for the risk stratification of thyroid nodule features on US scanning. Many of these systems use complex algorithms based on several US imaging features, which may be difficult to use depending on the experience of the individual performing the US scan. The aim of this study was to review the current evidence for US classification systems of thyroid nodules and their correlation to subsequent FNAB findings, with a view of providing suggestions for avenues for future research.

## Review

Methods

Searches

Recommendations from the Preferred Reporting Items for Systematic Reviews and Meta-Analyses were incorporated into this review [12]. Keywords included ‘ultrasound classification’, ‘thyroid nodules’, ‘fine needle aspiration’, and ‘malignancy’. Electronic searches were performed on PubMed and Scopus databases for English language studies published between September 2012 and September 2017. The search term used in each database were as follows: PubMed (ultrasound classification AND thyroid nodules AND fine needle aspiration AND malignancy); Scopus (TITLE-ABS-KEY [ultrasound classification AND thyroid nodules AND fine needle aspiration AND malignancy]). No filters for journal, study design, or subject were applied to the search, although conference proceedings and abstracts were excluded.

Study Selection

Two authors (Rakesh Mistry and Thushanth Sooriyamoorthy) independently assessed all studies from both searches against the eligibility criteria. Studies were included that identified the use of US imaging for the classification of thyroid nodules. The following studies were excluded in this study: studies with no full English text available/accessible and studies with no US-based system of classification of thyroid nodules and/or no attempt to correlate US findings with histology based on FNAB.

Data Extraction

Two authors (R.M. and T.S.) independently extracted data from the included studies into a self-designed template, referring to the Cochrane Handbook for Systematic Reviews of Interventions as a guide [13]. Study information and clinical characteristics were extracted from all studies (where reported), including the performance (sensitivity, specificity, positive predictive value [PPV], and negative predictive value [NPV]) of US imaging for the classification of thyroid nodules. Multiple authors (Christopher Hillyar, Anjan Nibber, R.M., and T.S.) evaluated the extracted data for accuracy and agreement.

Data Analysis

Not all studies reported all variables. Items that were not reported or were unclear were not included in the analysis. Data were analyzed using Microsoft Excel. Two authors (C.H. and A.N.) independently conducted data analysis.

Results

Study Characteristics

**Figure 1:**
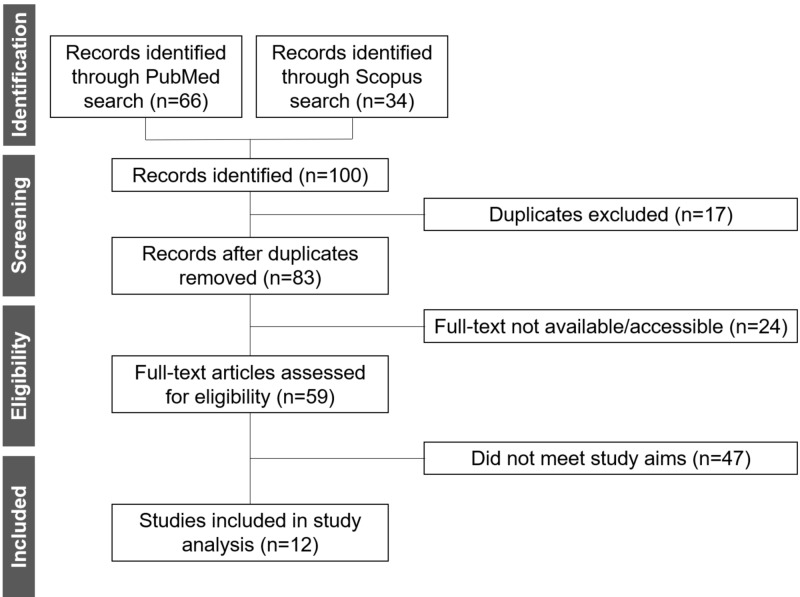
Identification of the eligible studies for analysis

The database searches identified 100 publications. After evaluation, 12 studies met the inclusion criteria (Figure 1). A summary of the results extracted from these studies is presented in Table 1.

**Table 1:** Summary of data extracted from the included studies TIRADS, Thyroid Imaging Reporting and Data System; ATA, American Thyroid Association; PPV, positive predictive value; NPV, negative predictive value; NR, not reported

Study type	Population characteristics			Nodule classification system	Ultrasound scan performance				Reference
	Participants (nodules); n	Male:female participants (nodules); n:n, % male	Mean/median age (range); years		Sensitivity (%)	Specificity (%)	PPV (%)	NPV (%)	
Guidelines	NR	NR	NR	TIRADS	88.0	49.0	49.0	88.0	Gharib et al. [14]
Multi-center study	3315 participants (3822 nodules)	NR (766:3056, 20% male)	Mean: 54 (18-97)	TIRADS	NR	NR	NR	NR	Middleton et al. [15]
Prospective study	2921 participants (3980 nodules)	951:1970, 33% male (NR)	Mean: 51 (16-78)	TIRADS	97.0	90.0	40.0	91.0	Zhang et al. [16]
Prospective study	238 participants (272 nodules)	NR (78:194, 29% male)	Patients with benign nodules, mean of 42 (NR); patients with malignant nodules, mean 43 (NR)	TIRADS	NR	NR	63.9	NR	Chandramohan et al. [17]
Prospective study	105 participants (NR)	15:90, 14% male (NR)	Median: 46 (16-80)	ATA guidelines	NR	NR	NR	NR	Rosário et al. [18]
Prospective study	100 participants (157 nodules)	14:86, 14% male (NR)	Mean: 49 (NR)	Bethesda	NR	NR	NR	NR	Puno-Ramos et al. [19]
Retrospective study	1241 participants (1293 nodules)	NR (NR)	Mean: 50 (18-87)	TIRADS vs ATA guidelines	97.4 vs 95.3	29.3 vs 37.4	23.3 vs 98.1	98.1 vs 97.3	Yoon et al. [20]
Retrospective study	906 participants (1000 nodules)	NR (NR)	NR (NR)	TIRADS	NR	NR	NR	NR	Rahal Jr et al. [21]
Retrospective study	100 participants (NR)	NR (NR)	NR (NR)	TIRADS	70.6	90.4	NR	93.8	Singaporewalla et al. [22]
Retrospective study	84 participants (87 nodules)	33:51, 18% male (NR)	Mean: 59 (34-85)	TIRADS	90.0	57.4	64.3	87.1	Yoon et al. [23]
Review	NR	NR (NR)	NR (NR)	Bethesda	NR	NR	NR	NR	Heller et al. [24]
Review	NR	NR (NR)	NR (NR)	NR	NR	NR	NR	NR	Papini et al. [25]

The included studies consisted mainly of prospective (n=4) and retrospective studies (n=4) followed by reviews/guidelines (n=3) and a multi-center study (n=1). In terms of population characteristics, four larges studies consisted of 3,315 participants (3,822 nodules), 2921 participants (3,980 nodules), 1,241 participants (1,293 nodules), and 906 participants (1,000 nodules) [15,16,20,21]. Five comparatively small studies included 238 participants (272 nodules), 105 participants (nodules not reported), 100 participants (157 nodules), 100 participants (nodules not reported), and 84 participants (87 nodules) [17-19, 22,23]. A total of 9,010 participants (10,611 nodules) were reported from all studies [14-25]. The proportion of participants who were male (nodules from male participants) reported from studies ranged from 14% to 33% (20-29%) [15-19,22,23]. The mean age of all participants was relatively consistent and reported to be 46-59 years [15,16,18-20,23]. One study reported the mean age only for participants with benign and malignant nodules: 42 and 43 years, respectively [17]. Another study reported the median age for all participants (46 years) [18]. By comparison, the reported age range varied to a greater extent, with a lower limit of 16-34 years and an upper limit of 78-97 [15,16,18,20,23].

Thyroid Nodule Classification Systems

In total, two US-based classification systems for thyroid nodules were identified: TIRADS and the American Thyroid Association (ATA) guidelines. In addition, the Bethesda classification system of histological reporting for FNAB of thyroid tissue was also reported. Eight (66%) studies reported the use of TIRADS [14-17,20-23] followed by two studies (8%) that used the ATA guidelines [18,20] and two (17%) studies that used the Bethesda system [19,24].

Performance of Ultrasound Imaging for the Classification of Thyroid Nodules

Of the eight studies using TIRADS, six reported performance parameters for US-based classification of thyroid nodules (e.g. sensitivity, specificity, PPV, and NPV) [14,16,17,20,22,23]. Of the two studies reporting on the ATA guidelines, only one included US imaging performance parameters [20]. No publications reported US imaging performance parameters with the Bethesda system. Table 2 summarizes the performance of US imaging for the classification of thyroid nodules using the TIRADS system.

**Table 2:** Performance of ultrasound-based classification of thyroid nodules using TIRADS PPV, positive predictive value; NPV, negative predictive value; TIRADS, Thyroid Imaging Reporting and Data System

Ultrasound performance parameter	Participants (nodules)	Median (%)	Range (%)	References
Sensitivity	4346 participants (5360 nodules)	90.0	70.6-97.4	[14,16,20,22,23]
Specificity	4346 participants (5360 nodules)	57.4	29.3-90.4	[14,16,20,22,23]
PPV	4484 participants (5632 nodules)	49.0	23.3-64.3	[14,16,17,20,23]
NPV	4346 participants (5360 nodules)	91.0	87.1-99.0	[14,16,20,22,23]

The sensitivity, specificity, PPV, and NPV ranged from 70.6% to 97.4%, 29.3% to 90.4%, 23.3% to 64.3%, and 87.1% to 99.0%, respectively. The median sensitivity reported for TIRADS was 90.0%, with three studies reporting a sensitivity for TIRADS of 90.0% or above [16,20,23]. Although one study reported that the sensitivity of TIRADS was 70.6%, this retrospective study assessed a small cohort of only 100 participants [22]. In contrast, analysis of the sensitivity of TIRADS from larger studies gave sensitives of 97.0%-97.4%, when a total of 5,273 nodules (from 4,162 participants) were assessed [16,20]. Due to a wide range of reported results, the median specificity (57.5%) for TIRADS was considerably poorer than the sensitivity. Only one large study assessing 3,980 nodules (from 2,921 participants) reported favorable results with a specificity of 90.0% [16], whereas two studies, including one assessing 1,293 nodules (from 1,241 participants), reported specificities of less than 50%, with the lowest specificity reported being 29.3% [14,20]. The median PPV (49.0%) for TIRADS was similarly poor, with two large studies including a total of 4,162 nodules (from 5,273 participants) reporting PPVs of 23.3%-40.0% [16,20]. Finally, the median NPV (91.0%) of TIRADS was excellent and was the most consistently reported performance parameter with the lowest range. Three of five studies reported NPVs of greater than 90.0% [16,20,22], with two of these being large studies including a total of 5,273 nodules (from 4,162 participants), which reported an NPV of 91.1%-98.1% [16,20].

In one study, a direct comparison was made between the TIRADS and the ATA guidelines [20]. Yoon et al. reported that TIRADS was superior to the ATA guidelines in terms of sensitivity (97.4% vs 95.3%; p<0.001), although the ATA guidelines were superior to TIRADS in terms of specificity (37.4% vs 29.3%; p<0.001) and PPV (98.1% vs 23.3%; p<0.001). No statistically significant difference was observed between TIRADS and the ATA guidelines in terms of NPV. This study also reported that, unlike TIRADS, some nodules could not be classified using the ATA guidelines [20].

## Conclusions

This study assessed the literature on US-based thyroid nodule classification systems, which demonstrated that TIRADS has utility at classifying thyroid nodules. The variability of the specificity of TIRADS, which was borne out by large studies assessing thousands of thyroid nodules, suggests that the performance of US, especially at classifying nodules as negative for disease, is highly dependent on the skill of the operator. In clinical practice, the poor PPV of TIRADS may be associated with an excess number of FNABs of benign nodules and represents a source of procedural risk, reduced cost-effectiveness, and unnecessary discomfort and concern for the patient. Although mild pain from FNAB can be controlled with paracetamol, future research should focus on quantifying the pain and stress encountered by patients undergoing FNAB for thyroid nodules. The favorable NPV of TIRADS may offset the impact of the PPV and help reduce the number of unnecessary FNABs of benign thyroid nodules. The paucity of studies assessing the ATA guidelines makes any comparison with TIRADS a tentative assessment at best and represents a significant opportunity for further research. Thus, research directed at improving the TIRADS system using powered studies with large patient populations is required to compare TIRADS with other classification systems (ATA guidelines/Bethesda) in order to demonstrate superiority. This may be used to inform and update the British Thyroid Association (BTA) guidelines, an area of particular importance in the UK.
